# Adaptive Evolution in Ecological Communities

**DOI:** 10.1371/journal.pbio.1001332

**Published:** 2012-05-15

**Authors:** Martin M. Turcotte, Michael S. C. Corrin, Marc T. J. Johnson

**Affiliations:** 1 Department of Biology, University of Toronto at Mississauga, Mississauga, Ontario, Canada; 2 Biomedical Communications Program, Department of Biology, University of Toronto at Mississauga, Mississauga, Ontario, Canada

## Abstract

Understanding how natural selection drives evolution is a key challenge in evolutionary biology. Most studies of adaptation focus on how a single environmental factor, such as increased temperature, affects evolution within a single species. The biological relevance of these experiments is limited because nature is infinitely more complex. Most species are embedded within communities containing many species that interact with one another and the physical environment. To understand the evolutionary significance of such ecological complexity, experiments must test the evolutionary impact of interactions among multiple species during adaptation. Here we highlight an experiment that manipulates species composition and tracks evolutionary responses within each species, while testing for the mechanisms by which species interact and adapt to their environment. We also discuss limitations of previous studies of adaptive evolution and emphasize how an experimental evolution approach can circumvent such shortcomings. Understanding how community composition acts as a selective force will improve our ability to predict how species adapt to natural and human-induced environmental change.

## Abstract 2

Multi-species interactions can influence evolution in ways that are unpredictable from studies that focus on simpler communities because direct and indirect species interactions alter the strength and direction of selection.

## A Brief History of the Study of Adaptive Evolution

Understanding how the diversity of life arose on Earth and what drives species to change through time is a problem that has fascinated scientists and the general public alike since before Darwin. Predicting evolution even within a single population remains a difficult challenge because a population can adapt to the abiotic environment (e.g., temperature), to another species with which it interacts (e.g., a competitor), or to a combination of these abiotic and biotic forces. Despite the difficulty of this challenge, a comprehensive understanding of evolution is imperative because it would allow scientists to predict whether species can adapt to rapid climate change [1], help conserve endangered species [2], design more effective treatments against infectious diseases and cancer [3],[4], and to control pests attacking agricultural crops [5]. Here we explore how the diversity of species within communities affects adaptive evolution to a changing environment.

Although Darwin himself applied empirical methods to study adaptive evolution throughout his work, it was not until the early 20th century that rigorous experimental methods were employed. The discipline known as “genecology” was among the first to focus on how populations adapt to abiotic factors such as climate and soil conditions. These studies typically involved large transplant experiments where individuals, collected from multiple locations differing in environmental conditions, were grown together in a common environment [6],[7]. This research showed that plant populations frequently exhibit genetically based differences in morphological, life-history, and physiological traits that make them better adapted to the environments from which they were collected.

In the 1960s, research shifted towards investigating the importance of ecological interactions among species in driving adaptive evolution [8],[9]. Early mathematical theory showed how interspecific competition could drive the evolution of character displacement (Box 1) in traits associated with resource use [9], which led to decades of empirical research on the topic [10]. Also during this time, the concept of coevolution (Box 1) was proposed as an important process that explained the diversity of plants and their enemies [8]. This led to an explosion of research focusing on pairwise coevolution where two interacting species impose reciprocal natural selection on one another, which drives evolution in both species [11]. Many of these studies identified species with congruent traits (e.g., a plant with a defensive compound and an herbivore with a detoxification enzyme) and attributed such observations to coevolution.

**Box 1. Glossary of Terms**

**Character displacement:** Traits evolve to become more dissimilar between interacting species. This can be caused by selection to reduce competition for limiting resources.

**Coevolution:** Evolution between a pair of interacting species in response to reciprocal natural selection on each other. If this coevolution is not influenced by the presence of interacting species, then it is said to be pairwise coevolution. Diffuse coevolution occurs when the strength or direction of selection between coevolving species, or the evolutionary response to selection, depends on the presence of other members in the community.

**Experimental evolution:** Experiments that manipulate the selective environment experienced by replicated populations and subsequently measure their evolutionary response over one to thousands of generations.

**Geographic mosaic theory of coevolution:** A framework that explores how spatial variation in the biotic and abiotic environment affects coevolution between two species [12].

**Niche:** The fundamental niche consists of the environmental conditions under which a species can persist, whereas the realized niche is a subset of those conditions where persistence is possible while sharing that environment with other species (e.g., competitor) [29],[30].

**Figure 1:**
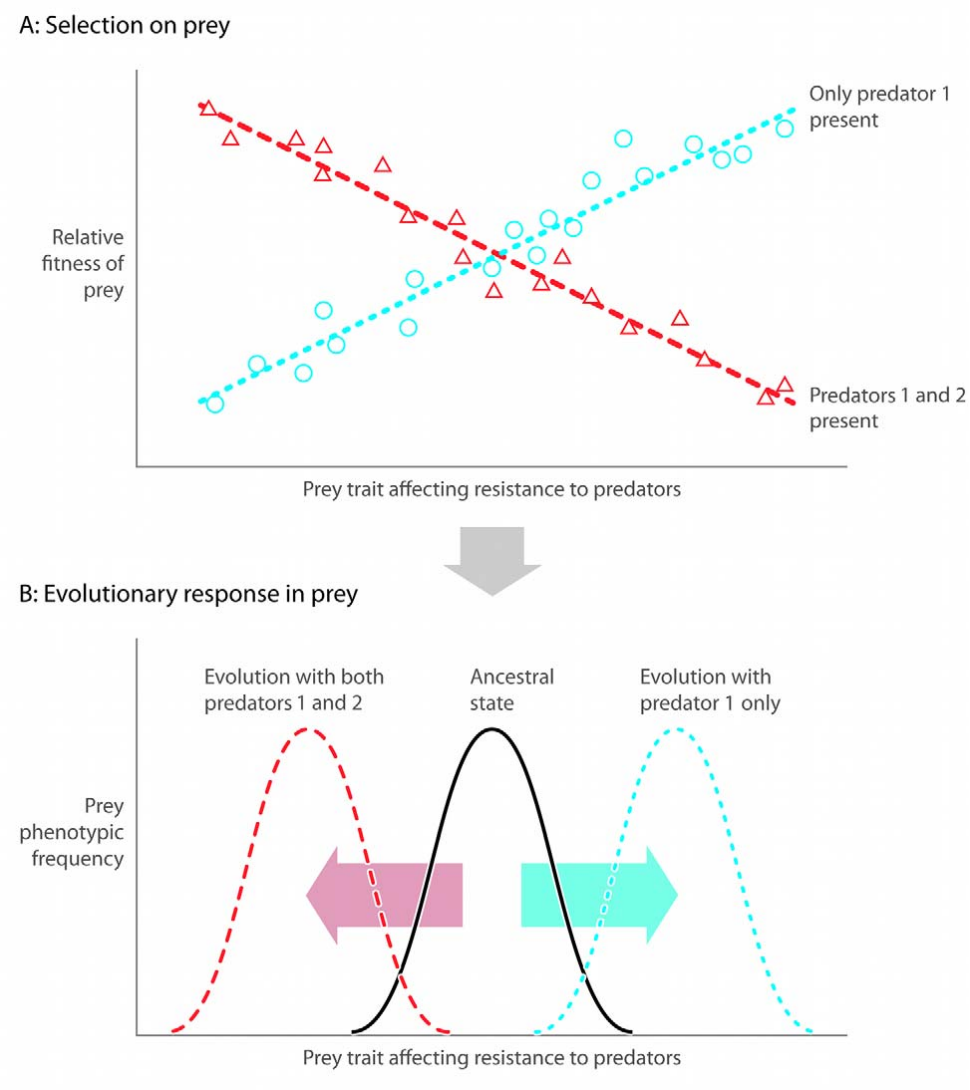
The effects of community complexity on natural selection and evolution. This figure presents a hypothetical case where increasing complexity in a predator–prey community alters natural selection and the evolutionary response of a single prey population. Panel (A) depicts the genetic covariance between relative fitness and a specific resistance trait in the prey, where the slope of the line is the strength of selection on the resistance trait. The blue dotted line and circles represent a simple community composed of a single prey and a single predator population. The red dashed line and triangles represent a more complex community composed of the same prey species but in the presence of two predator species. Panel (B) shows how the prey species evolves in response to these selective pressures caused by different predator communities. The black line represents the ancestral trait distribution, whereas the dotted and dashed lines represent trait distributions after selection in the simple and complex communities, respectively. Benkman and colleagues identified a similar situation occurring in Rocky Mountain lodgepole pine, which is fed upon by red crossbills and red squirrels [37]. In populations without squirrels, crossbills selected for and caused evolution of longer cones with thicker distal scales and more seeds per cone. When crossbills and squirrels were both present, squirrels imposed stronger selection on cone morphology, which caused the evolution of shorter cones with fewer seeds that had thinner distal scales but thicker basal scales. The presence versus absence of squirrels also altered selection by trees on crossbill bill morphology. Therefore, the presence of squirrels altered selection and coevolution between crossbills and pine trees.

Modern studies of coevolution highlight how this process can be modified by variation in the abiotic environment and by the presence or absence of other species [11],[12]. Theoretical models [13],[14] show that predictions made from simple two-species communities are often not sufficient to predict evolutionary dynamics in complex natural systems, because additional species might alter the strength and direction of selection (Figure 1) [15]. Such complexities led to new approaches to study coevolution. For example, the idea of diffuse coevolution argues that coevolution between interacting species is driven and/or modified by interactions with many members of the community [11]. Such coevolution occurs when selection for a specific trait in one species depends on the presence of other species in the community (Figure 1) [15],[16]. For example, a field experiment on ivyleaf morning glory found that selection for resistance to deer depends on the presence of insect herbivores [17]. Although similar studies remain rare, it is increasingly believed that accurate predictions of evolution depend on considering the joint influence of multiple species as opposed to pairwise coevolution [15],[18]. A complementary approach termed the geographic mosaic theory of coevolution (Box 1; [12]) was developed as a framework to explain why patterns of coevolution vary between populations. It argues that coevolution is an important driver of evolution but that temporal and spatial variation in abiotic and biotic factors influences the strength of coevolution. It asserts that to understand the coevolutionary process, scientists must study ecological and evolutionary processes over a broad geographic area.

## Limitations of Previous Studies of Adaptive Evolution

Recent findings suggest that evolution is affected by the diversity and composition of species within communities, but a seamless integration of community ecology and evolutionary biology remains elusive [18],[19]. One limitation of the methods developed to test predictions relating to diffuse coevolution is their focus on measuring selection over a single generation, instead of quantifying actual evolutionary change [15]–[17]. Selection over a single generation might not predict evolution because the strength and direction of selection can vary in time and space, gene flow of less fit alleles can overwhelm selection, and genetic correlations might impede a response to a given selective pressure. In addition, previous studies of coevolution focus on patterns in trait matching between species, which is often used to support the importance of past selection due to species interactions. This approach can be problematic because simulations show that spatial patterns in interspecific trait values used to support the geographic mosaic theory of coevolution can also be caused by simpler non-coevolutionary mechanisms [20],[21].

Understanding how ecological communities drive adaptation requires experimentally manipulating putative selective agents (i.e., species composition) and following the evolutionary response of populations over one to multiple generations. Applying the methods of experimental evolution (Box 1) will mitigate many of the limitations of previous approaches [22]. Such experiments need not be restricted to microorganisms. For example, Reznick et al. [23] introduced guppies into sections of streams with different predation regimes, thus modifying the selective environment, and tracked evolutionary changes in life-history traits. Turcotte et al. [24] manipulated the genotypic composition of aphid populations and tracked their evolution in the field. Rapid evolution of cold tolerance was experimentally demonstrated by introducing marine stickleback into freshwater ponds [25]. Moreover, one can design experiments that manipulate the abiotic environment and community composition simultaneously to quantify how these factors interact to affect evolution.

## Experimental Test of Evolution by Natural Selection in Complex Communities

In this issue of *PLoS Biology*, Lawrence et al.'s [26] study exemplifies the use of experimental evolution to test how community composition affects evolution. They created replicate bacterial communities composed of either a single species or a mixture of four species competing for the same resources. To understand the ecological mechanisms underlying the observed evolutionary patterns, they identified the concentrations of specific resources consumed and created by each species using sophisticated nuclear magnetic resonance spectroscopy.

**Figure 2:**
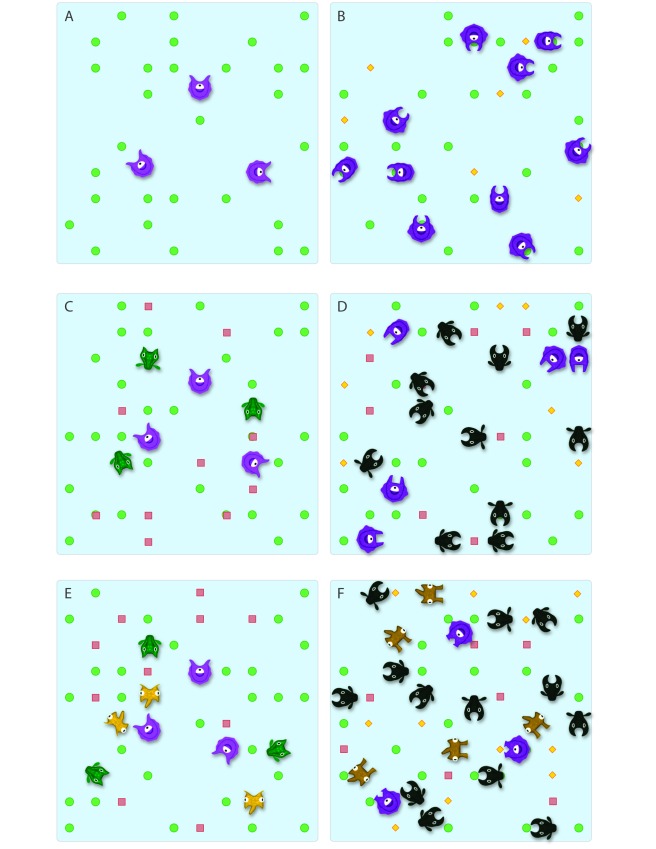
Evolutionary outcomes caused by differences in community composition. Cartoon examples of how community complexity can lead to unexpected ecological and evolutionary outcomes in populations. The illustrations are taken from a subset of idealized simulations that are depicted as animations in Movies 1–6. The panels on the left (A,C,E) represent initial conditions at the beginning of a simulation, and the panels on the right (B,D,F) show populations and communities after evolution has reached an equilibrium. In these examples, different consumer species (purple, green, and yellow) move in the environment consuming renewable resources (green circles and red squares; orange diamonds represent excrement). If they consume enough resources they reproduce, and if they do not they die. In all cases, species start as generalist consumers, represented here as non-specialized mouth parts capable of consuming any resource. Species can subsequently evolve to specialize on a resource by changing mouth shape to correspond to resource shape, which increases resource capture efficiency and reproduction. In reality, these examples apply to any case where a trait influences consumer efficiency, whether it involves morphological (e.g., beak morphology), physiological (e.g., metabolic rate), or behavioral (hunting method) change. (A) and (B) represent the evolution of specialization in a one species community. A single generalist species feeds on a common resource and evolves more efficient resource consumption (Movie 1). (C) and (D) represent the evolution of character displacement in a two-species community whereby two generalist consumer species initially compete for two limited resources. Competition causes each species to specialize on different resources and thus avoid extinction (Movie 3). (E) and (F) represent coexistence of three species that evolve to specialize on one of the two limited resources (green circles and red squares) or on the waste products produced by other species (orange diamonds) (Movie 6), as observed by Lawrence et al. [26].

Their results show that community complexity influences adaptive evolution in ways that would never have been predicted by single-species experiments. When species were grown individually, three species evolved faster reproduction after 60–70 generations compared to their non-evolved ancestors (see Figure 2A; Movie 1 in Additional Resources). When species grew together and competed for resources, one species evolved dramatically faster reproduction, while the other three species evolved slower reproduction. The authors' mechanistic investigation revealed that species interactions drove the evolution of alternative resource use not observed in single-species communities; one species appeared to specialize on complex carbon sources while another specialized on a less common sugar resource. Competition thus drove character displacement (Figure 2B, Movie 3). Surprisingly, the last two species avoided extinction by evolving the ability to consume different waste products produced by the other species (Figure 2C, Movie 6). Therefore, evolution changed the role of one species from a competitor to a facilitator because it provided resources that benefited another species that transitioned to a more commensalistic scavenging lifestyle (see movies for other possible outcomes).

These results demonstrate how multi-species communities can alter the environment experienced by other species and lead to a diversity of new and unpredictable evolutionary outcomes. In the study by Lawrence et al. [26], the unpredictable results are explained in part by evolution in some species to use waste products created by other species. Although this may seem like a peculiarity of bacteria, evidence in nature suggests such phenomena may be widespread. For example, some African cichlid fish species, which have undergone adaptive radiation, have evolved the ability to consume resources provided by other cichlids. These include species that consume other cichlids, others that eat eggs, and still others that consume fins and scales, and these behaviors have led to the evolution of morphological differentiation [27]. Other examples of how complex communities can influence evolution in unique ways include coevolved symbioses among multiple species, such as leaf cutter ants that show evidence of 50 million years of coevolution and cospeciation with as many as four other fungal and bacterial symbionts [28]. In general, certain adaptive pathways exhibited by species' populations would not exist if not for the presence of other species in the community.

The importance of studying adaptation in complex natural communities parallels research on the ecology and evolution of the niche (Box 1; [29],[30]). A species' fundamental niche includes the entire range of environmental conditions in which the species can persist in the absence of interactions with other species. In theory, one can predict a species' distribution with great accuracy based on knowledge of its fundamental niche. These predictions are bound to fail, however, when species interactions in communities alter the ecology and evolution of populations, such that the realized niche of a species can vary greatly from the fundamental niche.

Lawrence et al.'s [26] study is important because it provides rare experimental data revealing how community composition affects evolution. It implies that complex communities can drive evolution along multiple paths that might not be available in simpler communities. Complex communities can select for more efficient resource use, can alter which resource(s) is used, or affect diversification of lineages [31]. These results imply that predictions constructed from single-species experiments might be of limited use given that most species interact with many others in nature [18],[32].

Additionally, Lawrence et al. found that evolution can feedback to influence ecosystem functioning, an area of increasing interest in biology [33]. Communities created with bacteria that had evolved together had higher productivity than communities composed of bacteria that evolved in isolation. This occurred because interspecific competition led to the specialization of three bacterial species onto different resources, thus enabling higher productivity due to the evolution of increased complementarity. This result implies that over short time periods rapid evolution can drive ecological dynamics [34]. Therefore, understanding changes in ecosystem processes, such as productivity, might require one to consider not only changes in species composition, but also evolutionary changes in the properties of those species [35]. This result, however, was only apparent because they studied evolutionary change over multiple generations as opposed to a single generation of selection.

## Challenges and Future Directions

If evolutionary biology is to become a predictive science, future research needs to embrace the complexity inherent to communities and ecosystems. Although this idea has been applied in some modern applications of coevolution [12],[15], it is important that we now study the actual process of adaptation over multiple generations. In this regard it will be important to move beyond studying static patterns of trait variation and selection that are currently employed, which can provide a misleading snap-shot of evolution [20]. For example, Terhorst [32] found that predation and interspecific competition interact to affect the evolutionary dynamics of protozoans in the laboratory. The next step is to bring these experimental evolution methods to the field to understand what drives adaptation in nature [23],[36]. Although more difficult, experiments that manipulate the composition of complex communities and observe evolutionary dynamics are possible. For example, aphids evolved more rapidly in the presence of a natural community of enemies and competitors as opposed to when these were excluded [24]. The authors suspect that competitors are reducing resource availability for the aphids, which in turn increases intraspecific competition and the strength of clonal selection.

In conclusion, we argue that the study of adaptive evolution would benefit from experimental tests of how multi-species interactions affect evolution, as exemplified by Lawrence et al. [26]. The complexity of natural systems suggests that the importance of community composition will vary greatly in both time and space. Improving our ability to predict evolution will require us to quantify how different selective forces (e.g., temperature, community composition) shape evolutionary processes. Factorial evolution experiments in the lab and field, which manipulate multiple agents of natural selection, will provide the best way forward. Although addressing these challenges will be a difficult task, the effort is rich in rewards as it will ultimately make evolutionary biology a more predictive science and provide much needed insight into many applied problems.

## Additional Resources

The following six animations, all available at http://bmc.erin.utoronto.ca/evoeco/, illustrate how community complexity can cause different evolutionary outcomes. Movies 1, 3, and 6 are also summarized in Figure 2. These movies illustrate idealized scenarios highlighting a subset of potential evolutionary outcomes. 

**Movie 1**

**Evolution of specialization in a one-species community.** A single generalist species (Species A) feeds on a common resource and evolves more efficient resource consumption, which positively affects population growth rate.

**Movie 2**

**Evolution of specialization in a two-species community causes extinction of one competitor.** Two generalist consumer species initially compete for two limited resources. Species B evolves to specialize on consuming resource 1. Species A cannot support its population on resource 2 because that resource is less common and goes extinct.

**Movie 3**

**Evolution of character displacement caused by competition in a two-species community.** Two generalist consumer species initially compete for two limited resources. Competition causes each species to evolve to specialize on different resources and thus avoid extinction.

**Movie 4**

**Evolution of trophic structure in a two-species community.** Two generalist consumer species initially compete for two limited resources. Species B evolves to specialize on the more common resource, while Species A evolves the ability to consume species B. Thus, Species A evolves from being a competitor to a predator and both species are maintained via traditional predator–prey cycles.

**Movie 5**

**Evolution leads to coexistence among three consumers on two resources.** Three generalist species initially compete for two limited resources. Species B and Species C evolve to specialize on resources 1 and 2, respectively, whereas as Species A does not evolve and remains a generalist. This example illustrates the specific conditions whereby evolution of multiple consumers permits coexistence [38].

**Movie 6**

**Coexistence of three species due to evolution of specialization on waste products in a complex community.** Three generalist species initially compete for two limited resources. Species B and species C evolve to specialize on resource 1 and 2, respectively. Species A avoids extinction by evolving to specialize on the waste products generated by species 1 and 2. Therefore the presence of additional species creates new resources.
